# Heparin-Modified Collagen Gels for Controlled Release of Pleiotrophin: Potential for Vascular Applications

**DOI:** 10.3389/fbioe.2019.00074

**Published:** 2019-04-09

**Authors:** Francesco Copes, Pascale Chevallier, Caroline Loy, Daniele Pezzoli, Francesca Boccafoschi, Diego Mantovani

**Affiliations:** ^1^Laboratory of Human Anatomy, Department of Health Sciences, University of Piemonte Orientale, Novara, Italy; ^2^Laboratory for Biomaterials and Bioengineering, Canada Research Chair Tier I for the Innovation in Surgery, Department of Min-Met-Materials Engineering, CHU de Quebec Research Center, Laval University, Quebec, QC, Canada

**Keywords:** collagen, pleiotrophin, heparin, endothelial cells, smooth muscle cells, controlled release

## Abstract

A fast re-endothelialization, along with the inhibition of neointima hyperplasia, are crucial to reduce the failure of vascular bypass grafts. Implants modifications with molecules capable of speeding up the re-endothelialization process have been proposed over the last years. However, clinical trials of angiogenic factor delivery have been mostly disappointing, underscoring the need to investigate a wider array of angiogenic factors. In this work, a drug release system based on a type I collagen hydrogel has been proposed for the controlled release of Pleiotrophin (PTN), a cytokine known for its pro-angiogenetic effects. Heparin, in virtue of its ability to sequester, protect and release growth factors, has been used to better control the release of PTN. Performances of the PTN drug delivery system on endothelial (ECs) and smooth muscle cells (SMCs) have been investigated. Structural characterization (mechanical tests and immunofluorescent analyses of the collagen fibers) was performed on the gels to assess if heparin caused changes in their mechanical behavior. The release of PTN from the different gel formulations has been analyzed using a PTN-specific ELISA assay. Cell viability was evaluated with the Alamar Blue Cell Viability Assay on cells directly seeded on the gels (direct test) and on cells incubated with supernatant, containing the released PTN, obtained from the gels (indirect test). The effects of the different gels on the migration of both ECs and SMCs have been evaluated using a Transwell migration assay. Hemocompatibility of the gel has been assessed with a clotting/hemolysis test. Structural analyses showed that heparin did not change the structural behavior of the collagen gels. ELISA quantification demonstrated that heparin induced a constant release of PTN over time compared to other conditions. Both direct and indirect viability assays showed an increase in ECs viability while no effects were noted on SMCs. Cell migration results evidenced that the heparin/PTN-modified gels significantly increased ECs migration and decreased the SMCs one. Finally, heparin significantly increased the hemocompatibility of the collagen gels. In conclusion, the PTN-heparin-modified collagen here proposed can represent an added value for vascular medicine, able to ameliorate the biological performance, and integration of vascular grafts.

## Introduction

Every year is estimated that around 17.9 million of people die of cardiovascular diseases, mainly heart attacks and strokes, making them the first cause of death in the world (WHO^1^). Most of the time the root of these diseases can be found in atherosclerosis, a progressive pathology in which a plaque made of lipids, cholesterol, foamy cells, cellular debris, and calcium builds up in the walls of the arteries (Association^2^). Overtime, this plaque hardens and narrows the lumen of the affected artery, reducing the blood flow and ultimately leading to the aforementioned conditions. Despite the advances in pharmacological treatment and minimally-invasive surgical treatment, vascular bypass surgery remains the treatment of choice for atherosclerosis (Chlupác et al., 2009). The gold standard is the use of autologous vessels, such as saphenous vein, internal mammary arteries, and radial artery (Martínez-González et al., 2017). However, these options are not always available because of patients' condition. For these reasons, over the last 50 years surgeons reverted to the use of synthetic graft, such as polyethylene terephthalate (Dacron) or expanded polytetrafluoroethylene (ePTFE) based substitutes, instead of autologous vessels (Ravi and Chaikof, 2010). Despite being widely used in the clinical practice, the use of this substitute is still hampered by a high rate of graft failure, especially for small diameter vessels (Ø < 6 mm) (Chester, 2002). The main reasons of the grafts failure are intra-graft thrombosis, occurring in the first month after the implantation, and intimal hyperplasia, arising in the chronic phase especially at the anastomotic site (Bush, 1989). Incomplete healing process of the graft's, especially the lack of endothelialization, are the main causes of these two outcomes. The formation of a functional endothelial cells (ECs) layer is of crucial importance to avoid complications and to obtain an optimal integration of the implanted graft. Modification of the luminal surface with pro-endothelialization factors has been proposed over the years to help and increase ECs adhesion and proliferation, both alone (Greisler, 1996; Avci-Adali et al., 2011; Adipurnama et al., 2016) and in tandem with extracellular matrix (ECM) proteins (Steffens et al., 2004; De Visscher et al., 2012). However, clinical trials of pro-endothelialization enrichment of vascular grafts have been mostly unsatisfactory, thus the need to investigate new approaches (Springer, 2006) for this application.

In this work, a release system based on a type I collagen hydrogel (Wallace and Rosenblatt, 2003) has been proposed for the controlled release of pleiotrophin (PTN) (Rojas-Mayorquín and Ortuño-Sahagún, 2017), a 168 amino acids secreted cytokine known for its involvement in different cellular processes like cell growth and cell motility and for the beneficial effects exerted on the cardiovascular system (Deuel et al., 2002; Christman et al., 2005). Moreover, PTN has been recently shown to be able to exert potent pro-angiogenic factor on ECs compared to already used pro-endothelialization factors (Copes et al., 2019). Type I collagen is widely chosen as a biomaterial for medical applications due to its ease of extraction, weak antigenicity, robust biocompatibility, and its ability to be physically and chemically modified for a variety of applications (Lee et al., 2001; Gelse et al., 2003; Lynn et al., 2004). Due to its favorable properties, collagen-based matrices have been thoroughly investigated as a releasing system for therapeutic drug delivery applications (Bradley and Wilkes, 1977; Wallace and Rosenblatt, 2003; Koch et al., 2006; Chiu et al., 2010). Drug delivery systems have been widely studied in the last years. These systems utilize specific non-covalent interactions to stabilize drugs and immobilize them within a biocompatible matrix, thus protecting their biological activity and slowing their diffusion from the matrix. One of such drug delivery systems are heparin-based delivery systems (Sakiyama-Elbert, 2014). Heparin is a polysaccharide made up of repeating disaccharides (Rabenstein, 2002) best known for its anticoagulant properties, but has also been shown to promote cell adhesion, inhibit smooth muscle cell proliferation and to moderate inflammation (Young, 2008). Moreover, heparin is also known to sequester, stabilize and protect growth factors and cytokines and has been widely used in conjunction with different scaffolds to enhance their retention ability (Sakiyama-Elbert, 2011). In virtue of this favorable properties and in light of its high binding affinity for PTN (Rauvala, 1989), heparin has been used to better control the release of PTN from the collagen gel. The biological performances of the PTN-based drug delivery system have been investigated on both ECs and smooth muscle cells (SMCs).

## Materials and Methods

### Collagen Gels Preparation

Type I collagen was extracted from rat tails tendons and subsequently solubilized in 0.02 N acetic acid, as previously reported (Habermehl et al., 2005; Boccafoschi et al., 2007), to obtain a final collagen concentration of 4 g/L. For the preparation of the collagen gel, the collagen solution has been mixed with a buffer solution containing Dulbecco's modified Eagle medium (DMEM, Gibco, Invitrogen Corporation, Burlington, ON, Canada, 1.1X), NaOH (15 mM), and HEPES (20 mM) in deionized water to adjust the pH of the final solution and to initiate the polymerization process. P/S-M199 was then added to complete the basic composition of the control collagen gel (CTRL Gel). For the heparin-modified collagen gels, heparin sodium salt (Sigma Aldrich, Oakville, ON, Canada) has been added to the M199 portion of the collagen gel mix to obtain a final concentration of 10 μg/ml (H10 Gel). Heparin concentration has been chosen based on gelification analysis results of three different concentrations: 10, 25, and 50 μg/ml. With the concentration of 25 and 50 μg/ml the gelification of collagen was incomplete, thus the decision to work with the 10 μg/ml concentration. For the PTN-modified collagen gels, recombinant human PTN (Sigma Aldrich, Oakville, ON, Canada) has been added to the M199 portion of the collagen gel mix to obtain a final concentration of 150 ng/ml (P150 Gel). For the heparin-PTN-modified collagen gels, both heparin and PTN have been added to the M199 portion of the collagen gel mix to obtain the aforementioned concentration (H/P Gel). The concentrations used for the heparin and PTN have been chosen following a dose-response curve obtained in the preliminary steps of the study (data not shown). All the blends for the different experimental condition have been carefully mixed and 500 μl of the different solutions were poured into 24 wells culture plates and let gelify at room temperature (RT) for 1 h. Once jellified, collagen gels have been used for the subsequent experiments. Volumes used for the preparation of all the collagen gels formulation are shown in Table 1.

**Table 1:** Volume used for the preparation of the Collagen gels.

	**Gel volume**	**Collagen [4 g/l]**	**Buffer solution**	**P/S-M199**	**Heparin [5 mg/ml]**	**PTN [1μg/ml]**
CTRL Gel	500	250	125	125	X	X
H10 Gel	500	250	125	124	1	X
P150 Gel	500	250	125	50	X	75
H/P Gel	500	250	125	49	1	75

*Volume are shown in μl. Buffer Solution: Dulbecco's modified Eagle medium (1.1X), NaOH (15 mM), and HEPES (20 mM) in deionized water*.

### Unconfined Stress/Relaxation Compression Mechanical Tests

Stress/relaxation unconfined compression tests were performed on CTRL gel and H10 gels to evaluate possible changes in the mechanical properties due to the addition of heparin to the gel mix. Briefly, CTRL and H10 gels were prepared and after 24 h they have been placed in the chamber of a MACH-1 Mechanical Testing System (Biomomentum Inc., Laval, QC, Canada). Tests were performed in a bath containing PBS 1X at room temperature. The relaxation test consisted of compressing the sample according to the following parameter: Ramp amplitude (mm) = 5% of initial sample thickness; Ramp velocity (mm/s) = 5% of initial sample thickness; Number of ramp = 5; Fixed relaxation time (s) = 1,500 s. The relaxation time was defined in order to consider the viscoelastic behavior of the collagen gels and to reach a steady value for the load (equilibrium stress). The stress was recorded as a function of time. Following the stress/relaxation, the data obtained have been analyzed using MATLAB software (MathWorks, Natick, MA, USA) considering equilibrium strains and using the linear portion of the stress-strain curve at 15% of strain to obtain the equilibrium elastic modulus of the different gel formulations.

### Immunofluorescence

For immunofluorescence, CTRL and H10 gels have been prepared as already mentioned. After 24 h, gels have been incubated in PBS 1X with 3% of bovine serum albumin (BSA, Sigma Aldrich, Oakville, ON, Canada) for 10 min. Then gels have been incubated with mouse primary antibody for collagen type 1 (1: 1,000; Novus Biological, Oakville, ON, Canada) for 2 h at 37°C. Following, gels were incubated with an Alexa Fluor® 488 goat anti-mouse secondary antibody (Life Technologies, Sigma Aldrich, Oakville, ON, Canada) for 2 h at room temperature under agitation. Afterwards, gels have been rinsed three time with PBS 1X with 0.01% Tween 20 and have been kept overnight at 4°C before being placed on fluorescent microscope slides. Images at a magnification of 20X have been collected using an Olympus BX51 Fluorescence Microscope (Olympus Canada Inc., Toronto, ON, Canada).

### Cell Isolation and Culture

Human umbilical vein endothelial cells (HUVECs) and human umbilical artery smooth muscle cells (HUASMCs) were used in this study. Cells were isolated from human umbilical cord samples obtained from normal term pregnancies. Written informed consent was obtained from all mother donors according to the Declaration of Helsinki. All experiments were performed in compliance with the Canadian Tri-Council Policy Statement: Ethical Conduct for Research Involving Humans and institutional CHU de Quebec—Laval University guidelines. The protocol was approved by the Ethics Committee of the CHU de Quebec Research Centre (CER #S11-03-168). Briefly, umbilical cord samples, ~15 cm in length, were collected in phosphate-buffered saline solution (PBS, Fisher Scientific, Fair Lawn, NJ, USA) supplemented with 5% penicillin/streptomycin (P/S, Gibco, Invitrogen Corporation, Burlington, ON, Canada), solution to avoid any contamination and maintained at 4°C until processing.

As previously described (Loy et al., 2016), for HUVECs isolation, veins were rinsed with PBS, filled with 10× trypsin–EDTA solution (Gibco, Invitrogen Corporation, Burlington, ON, Canada), and incubated for 15 min at 37°C, after which the trypsin–EDTA solution containing the HUVECs was collected. PBS was added to wash the lumen, collected along with the previous solution, and centrifuged at 1,000 rpm for 5 min. Thereafter, the supernatant was removed and the cells resuspended in M199 culture medium (Gibco, Invitrogen Corporation, Burlington, ON, Canada) with 5% fetal bovine serum (FBS, Gibco, Invitrogen Corporation, Burlington, ON, Canada), 1% P/S (Gibco, Invitrogen Corporation, Burlington, ON, Canada), 2 ng/ml fibroblast growth factor (FGF, Life Sciences, Grand Island, NY, USA), 1 ng/ml endothelial growth factor (EGF, Life Sciences, Grand Island, NY, USA), 1 μg/ml ascorbic acid (Sigma Aldrich, Oakville, ON, Canada), 1 μg/ml hydrocortisone (Sigma Aldrich, Oakville, ON, Canada) and seeded in a 75 cm^2^ flask (Corning, Oneonta, NY, USA). This medium, that will be referred to as complete HUVECs M199 culture medium (HUVEC-M199), has been used in the experiments along with a basic version containing P/S (P/S-M199) only. Culture medium was changed after 24 h and then every 48 h until confluence was reached. ECs were characterized using a rabbit primary antibody against von Willebrand factor (VWF, Abcam, Ab6994, dilution 1/100, Toronto, ON, Canada, data not shown). The cells were then maintained in culture at 37°C in a saturated atmosphere at 5% CO_2_. When 85–90% of confluence was reached, cells were then enzymatically detached from the plate (0.05% trypsin, Gibco, Invitrogen Corporation, Burlington, ON, Canada) and then reseeded at a ratio of 1:3 or used for experiments. For the experiment here reported, cells have been used at passage 5 and 6.

For the HUASMCs, once all the associated connective tissues were carefully removed, arteries were cut open longitudinally. The intima layer, composed of endothelial cells, was carefully scraped off and the arteries were then cut in smaller pieces using a scalpel. The pieces were then placed in Petri dishes in presence of M199 culture medium (Gibco, Invitrogen Corporation, Burlington, ON, Canada) additioned with 5% fetal bovine serum (FBS, Gibco, Invitrogen Corporation, Burlington, ON, Canada), 1% penicillin/streptomycin (P/S, Gibco, Invitrogen Corporation, Burlington, ON, Canada), 2 ng/ml fibroblast growth factor (FGF, Life Sciences, Grand Island, NY, USA), 1 ng/ml endothelial growth factor (EGF, Life Sciences, Grand Island, NY, USA), 1 μg/ml ascorbic acid (Sigma Aldrich, Oakville, ON, Canada), 1 μg/ml hydrocortisone (Sigma Aldrich, Oakville, ON, Canada), and 5 μg/mL of human insulin solution (Santa Cruz Biotechnology, Dallas, TX, USA). This medium, that will be referred to as complete HUASMCs M199 culture medium (HUASMC-M199), has been used in along with P/S-M199). After 2 weeks, SMCs from the explants had migrated and colonized the surface of the Petri dishes. Once the artery pieces have been removed, cells were expanded in HUASMC-M199. Culture medium was changed every 48 h until confluence. SMCs were identified by immunostaining for smooth muscle-α-actin (SM-α-actin) and calponin (Ab7817, dilution 1/200 and Ab46794, dilution 1/200, Abcam, Toronto, ON, Canada) (data not shown,). Again, cells were maintained in culture at 37°C in a saturated atmosphere at 5% CO_2_. When 85–90% of confluence was reached, cells were enzymatically detached from the plate (0.05% trypsin, Gibco, Invitrogen Corporation, Burlington, ON, Canada) and then reseeded at a ratio of 1:3 or used for experiments. For the reported experiments, cells have been used at passage 7.

### Immunofluorescence

For immunofluorescence, CTRL and H10 gels have been prepared as already mentioned. After 24 h, gels have been incubated in PBS 1X with 3% of bovine serum albumin (BSA, Sigma Aldrich, Oakville, ON, Canada) for 10 min. Then gels have been incubated with mouse primary antibody for collagen type 1 (1:1,000; Novus Biological, Oakville, ON, Canada) for 2 h at 37°C. Following, gels were incubated with an Alexa Fluor® 488 goat anti-mouse secondary antibody (Life Technologies, Sigma Aldrich, Oakville, ON, Canada) for 2 h at room temperature under agitation. Afterwards, gels have been rinsed three time with PBS 1X with 0.01% Tween 20 and have been kept overnight at 4°C before being placed on fluorescent microscope slides. Images at a magnification of 20X have been collected using an Olympus BX51 Fluorescence Microscope (Olympus Canada Inc., Toronto, ON, Canada).

### Conditioned Medium Collection

After gelification, 600 μl of P/S-M199 has been added to each experimental condition. After 1, 3, and 7 days of incubation, medium has been completely removed and collected for subsequent experiment. For the ELISA quantification, additional time points at 10 and 14 days were added. At each time point, 600 μl of fresh P/S-M199 medium has been added to the gels.

### ELISA Quantification

For the quantification of the amount of PTN released by the different collagen gels preparation, an enzyme-linked immunosorbent assay (ELISA) was applied. Examination of PTN was done by RayBio® Human Pleiotrophin ELISA kit (RayBiotech, Norcross, Georgia, USA). The assay was performed according to the protocol provided by the manufacturer. Absorbance at a wavelength of 450 nm was recorded using a SpectraMax i3x Multi-Mode Plate Reader (Molecular Devices, San Jose, California, USA).

### Indirect Viability Assay

The effect of the released PTN on cells viability have been analyzed using an indirect viability assay performed on both HUVECs and HUASMCs. Briefly, cells have been seeded at a concentration of 20,000 cells/cm^2^ in 96 well culture plates. After 24 h of incubation with HUVEC-M199 or HUASMC-M199, depending on the cell type used, at 37°C in a saturated atmosphere at 5% CO_2_ to allow the adhesion of the cells, media has been removed and cells have been incubated for 24 h in presence of the different conditioned media collected as previously described. Cells cultivated in P/S-M199 medium have been used as a positive control (CTRL Cell). After the treatment, the conditioned media have been removed and cells have been incubated with a resazurin solution for 4 h. After the incubation, the highly fluorescent resorufin product obtained by the reduction of the resazurin was collected and fluorescence intensity at a 545 nm_ex_/590 nm_em_ wavelength was measured with a SpectraMax i3x Multi-Mode Plate Reader (Molecular Devices, San Jose, California, USA). Fluorescence intensity is proportional to cell viability. Data has been normalized toward the CTRL Cell condition.

### Direct Viability Assay

The effect of the addition of heparin and PTN to the CTRL gel formulation on cells viability has been analyzed using a direct viability assay performed using both HUVECs and HUASMCs. Briefly, the following collagen gels formulations have been prepared: (1) CTRL gel; (2) H10 gel; (3) P150 gel; and (4) H/P gel. After gelification, cells have been seeded in HUVEC-M199 or HUASMC-M199, according to the cell type, at a concentration of 20,000 cells/cm^2^ onto the different gels and incubated at 37°C in a saturated atmosphere at 5% CO_2_. Cells cultivated on culture polystyrene in HUVEC-M199 or HUASMC-M199 medium have been used as a positive control (CTRL Cell). After 1, 3, and 7 days two, media has been removed and cells have been incubated for six with a resazurin solution. After the incubation, the resorufin product obtained was collected and fluorescence intensity at a 545 nm_ex_/590 nm_em_ wavelength was measured with a SpectraMax i3x Multi-Mode Plate Reader (Molecular Devices, San Jose, California, USA). Fluorescence intensity is proportional to cell viability.

### Migration Assay

To test the effects of the released PTN on the migration of HUVECs, the transwell migration assay was used. Seven thousand and five hundred cells were seeded in the upper compartment of 24 well-format transwell with 8 μm pores (Corning, Amsterdam, the Netherlands) in 250 μL of HUVEC-M199 or HUASMC-M199, according to the cell type. In the lower compartment, the following collagen gels formulations have been prepared: (1) CTRL gel; (2) H10 gel; (3) P150 gel; and (4) H/P gel and 600 μl P/S-M199 have been added. Cells were incubated at 37°C in a saturated atmosphere at 5% CO_2_ for 24 h. After the incubation, cells on both faces of the insert membranes were fixed by incubation with formaldehyde 3.7% for 20 min at room temperature. Then, cells were stained with 1% Crystal Violet for 20 min at room temperature. Once stained, cells on the upper side of the porous membranes were gently removed using a cotton swab. The transwell inserts were then placed under a phase-contrast microscope and images of different fields (*n* = 5) were collected at 20X magnification. To assess the migration rate for each condition, stained cells were counted.

### Hemocompatibility Assay

To study the hemocompatibility of the different collagen gel formulations, the hemoglobin free methodology was used (Montaño-Machado et al., 2015). Briefly, the following collagen gels formulations have been prepared: (1) CTRL gel; (2) H10 gel; (3) P150 gel; and (4) H/P gel. After 24 h, 100 ml of citrated blood were deposited onto the surfaces of the different collagen gels and 20 μl of 0.1 M CaCl_2_ (Sigma Aldrich, Oakville, Canada) were immediately added to inhibit the anti-coagulant effect of the citrate. Samples were incubated at 37°C in a saturated atmosphere at 5% CO_2_ and after 10, 25, and 50 min, 2 ml of distilled water were added to each sample. The erythrocyte not entrapped in a blood clot were hemolyzed. One minute later, the obtained solution was removed and placed into a 96 well plate. The free hemoglobin molecules released in water following hemolysis were measured by reading the absorbance at a 540 nm wavelength by means of a SpectraMax i3x Multi-Mode Plate Reader (Molecular Devices, San Jose, California, USA). The higher is the absorbance recorded, the higher is the amount of free hemoglobin, therefore the higher is the hemocompatibility. The test was performed, with blood from different donors used for each experiment. The maximum amount of hemoglobin (Max Hemoglobin) was obtained by immediately hemolyzed after the citrate inhibition. Data where normalized toward the Max Hemoglobin value.

### Statistical Analysis

For each experiment, a *n* = 5 replicates for each condition has been used. Each of the experiments were performed three independent times. For the hemocompatibility test, blood from three different donors was used for each experiment. The data shown are means ± standard deviation (SD). Statistical significance of the presented results was calculated using ANOVA non-parametric Kruskal-Wallis method through the software InStat™ (GraphPad Software, La Jolla, CA, USA). Values of *p* < 0.05 or less were considered significant.

## Results

### Mechanical and Structural Characterization

**Figure 1:**
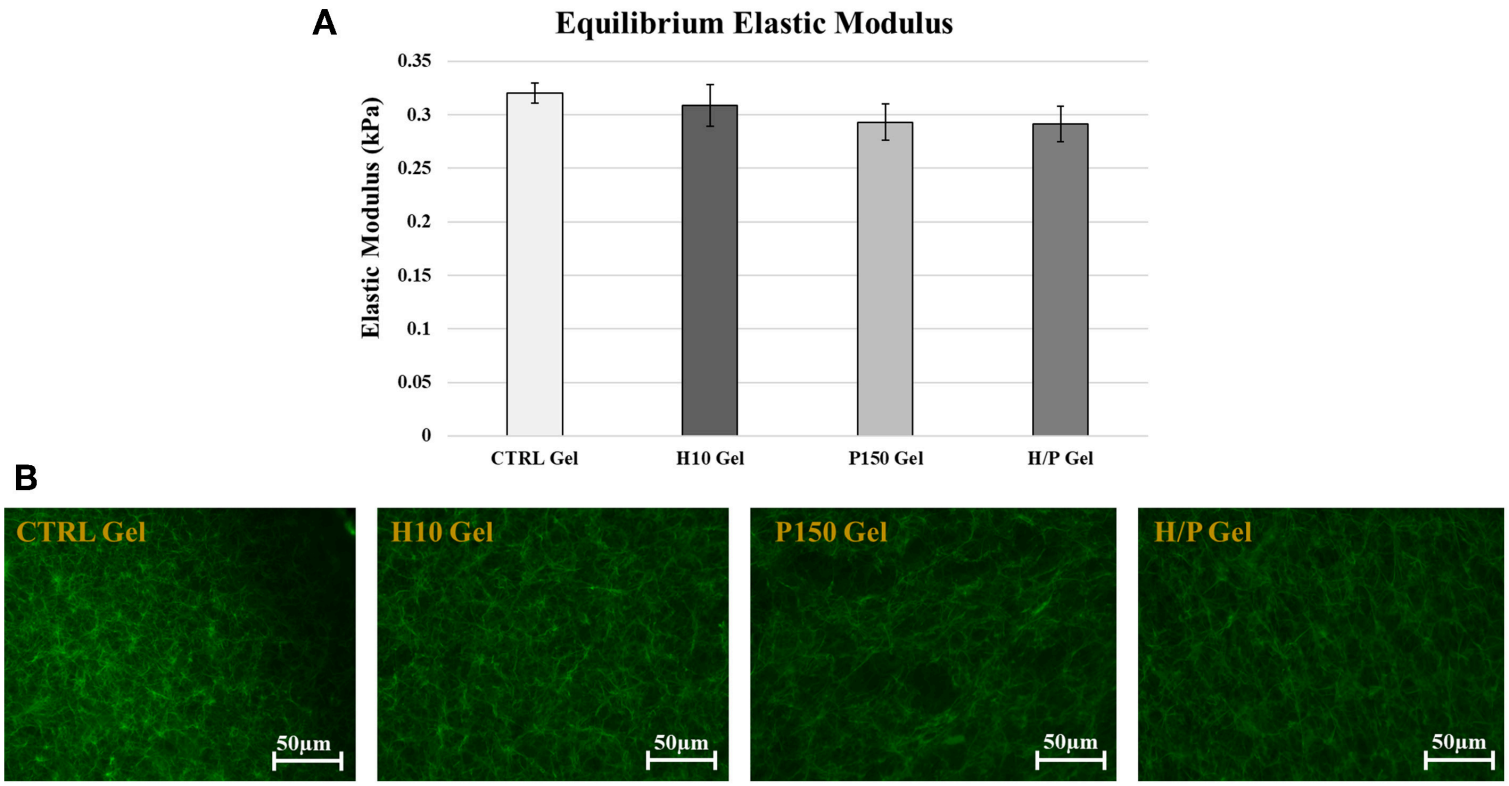
Mechanical and Structural Characterization. **(A)** Graph shows the mean Equilibrium Elastic Modulus ± SD for the 4 gel formulations: CTRL, H10, P150, and H/P gels. **(B)** The images show the immunofluorescent staining of the type 1 collagen fibers (green color) in the gel formulations tested: CTRL, H10, P150, and H/P gels. Images were taken after 24 h at a 20X magnification.

The stress/relaxation unconfined compression tests were performed on CTRL gels and on the modified gels to evaluate if the addition of heparin or PTN to the gel mix caused any changes in the mechanical properties of the gels. The equilibrium elastic modulus of the four gels composition has been analyzed: no significant differences in the equilibrium elastic modulus of the gel compositions were detected (Figure 1A). The immunofluorescence performed on the gels confirmed the results obtain by the mechanical characterization. In fact, no visible differences were noted in the arrangement of the collagen fibers in all the tested formulations of the gels (Figure 1B).

### Released PTN Quantification

**Figure 2:**
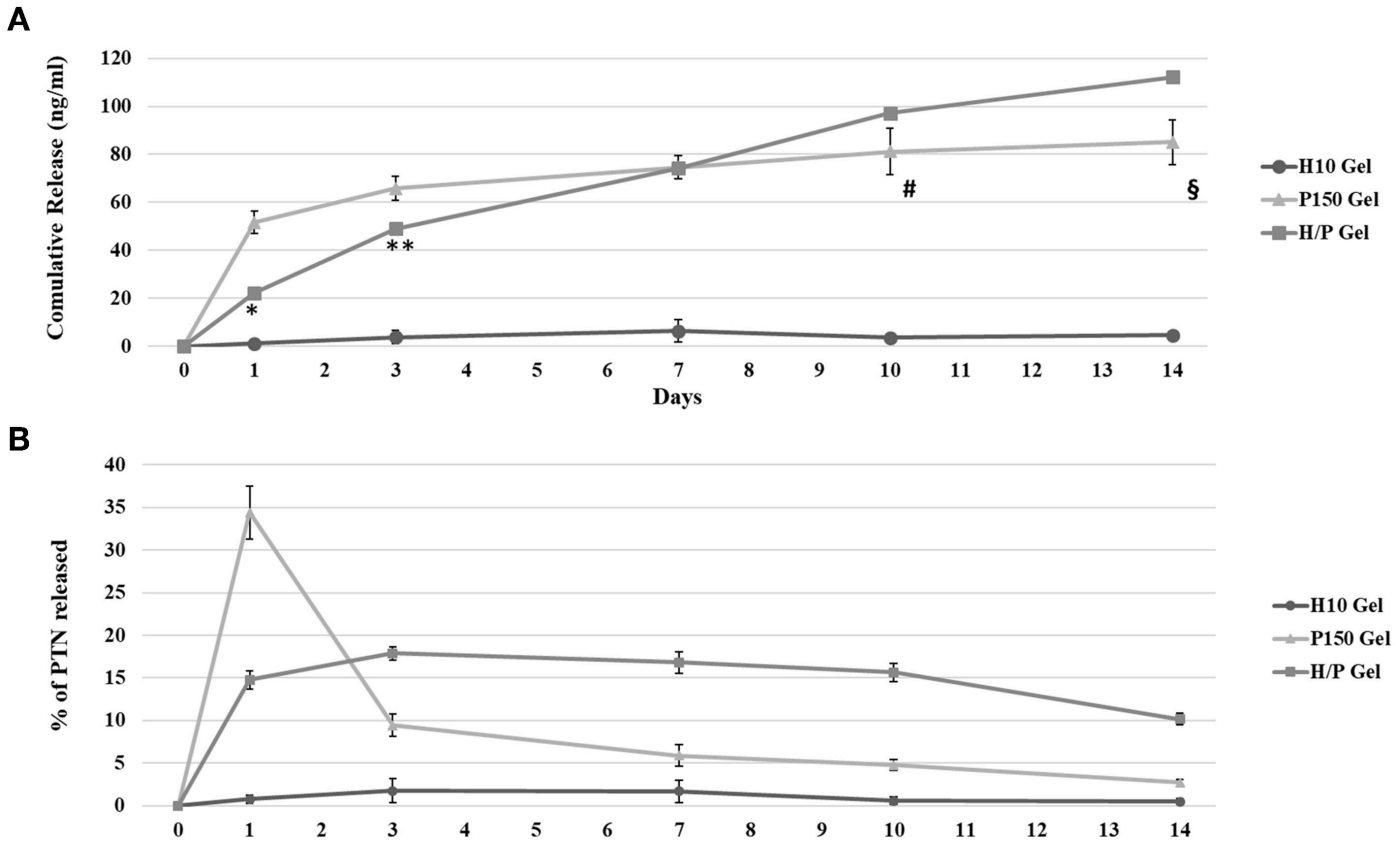
PTN ELISA Quantification. The graphic shows the results for the quantification of PTN release by the Hep 10 μg/ml (H10), PTN 150 ng/ml (P150) and Hep 10 μg/ml PTN 150 ng/ml (H/P) collagen gels after 1, 3, 7, and 10 days of incubation. **(A)** The graphic shows the mean cumulative release ± SD measured at each time point. ^*^p < 0.001 vs. 1 Day P150; ^**^p < 0.01 vs. 3 Days P150; ^#^*p* < 0.01 vs. 10 Days P150; ^§^*p* < 0.01 vs. 14 Days P150 gel. **(B)** The graphic shows the % of released PTN ± SD measured at each time point.

The amount of PTN released over a 10-days period, along with the kinetic of its release, has been analyzed by means of a PTN-specific ELISA quantification assay (Figure 2).

The results of the ELISA quantification have been used to analyses the cumulative released of PTN over 14 days, as shown in Figure 2A. For P150, after 1 day, the concentration of PTN found in the collected conditioned medium was 51.6 ± 4.7 ng/ml. After 3 days, the total PTN concentration detected was 65.8 ± 4.9 ng/ml and after 7 days the concentration was 74.6 ± 4.8 ng/ml. At 10 days, the cumulative released PTN was 81.2 ± 9.5 ng/ml wile at 14 days it reached 85.0 ± 9.2 ng/ml. A similar cumulative release was observed for the H/P gel, but the concentrations of PTN measured at the 1 and 3-days time points were significantly lower compared to the P150 condition (Day 1: 22.2 ± 1.5 ng/ml, *p* < 0.001 vs. P150 gel; Day 3: 49.0 ± 2.1 ng/ml, *p* < 0,01 vs. P150 gel). At 7 days, the cumulative release for the H/P gel, 74.3 ± 2.3 ng/ml, was almost the same as for the P150. However, at Day 10 the cumulative released PTN was higher compared to the P150 gel (97.3 ± 1.5 ng/ml, *p* < 0.01 vs. P150 gel). This trend continued until Day 14, with the cumulative release of H/P (112.3 ± 0.1 ng/ml) being higher than the P150 condition (*p* < 0.01 vs. P150 gel). Figure 2B shows the release kinetic of the different gel formulation expressed as % of released PTN at each time point studied. It is possible to observe how the PTN 150 ng/ml gel released a high amount of PTN at the first day while, during the following time points, the amount of released PTN decreased drastically. On the contrary, the amount of PTN released by the H/P gel was constant over time.

### Indirect Viability Test

Indirect viability tests have been performed on ECs and SMCs to evaluate if the released PTN present in the collected conditioned medium was able to exert any effect on the viability of the treated cells.

**Figure 3:**
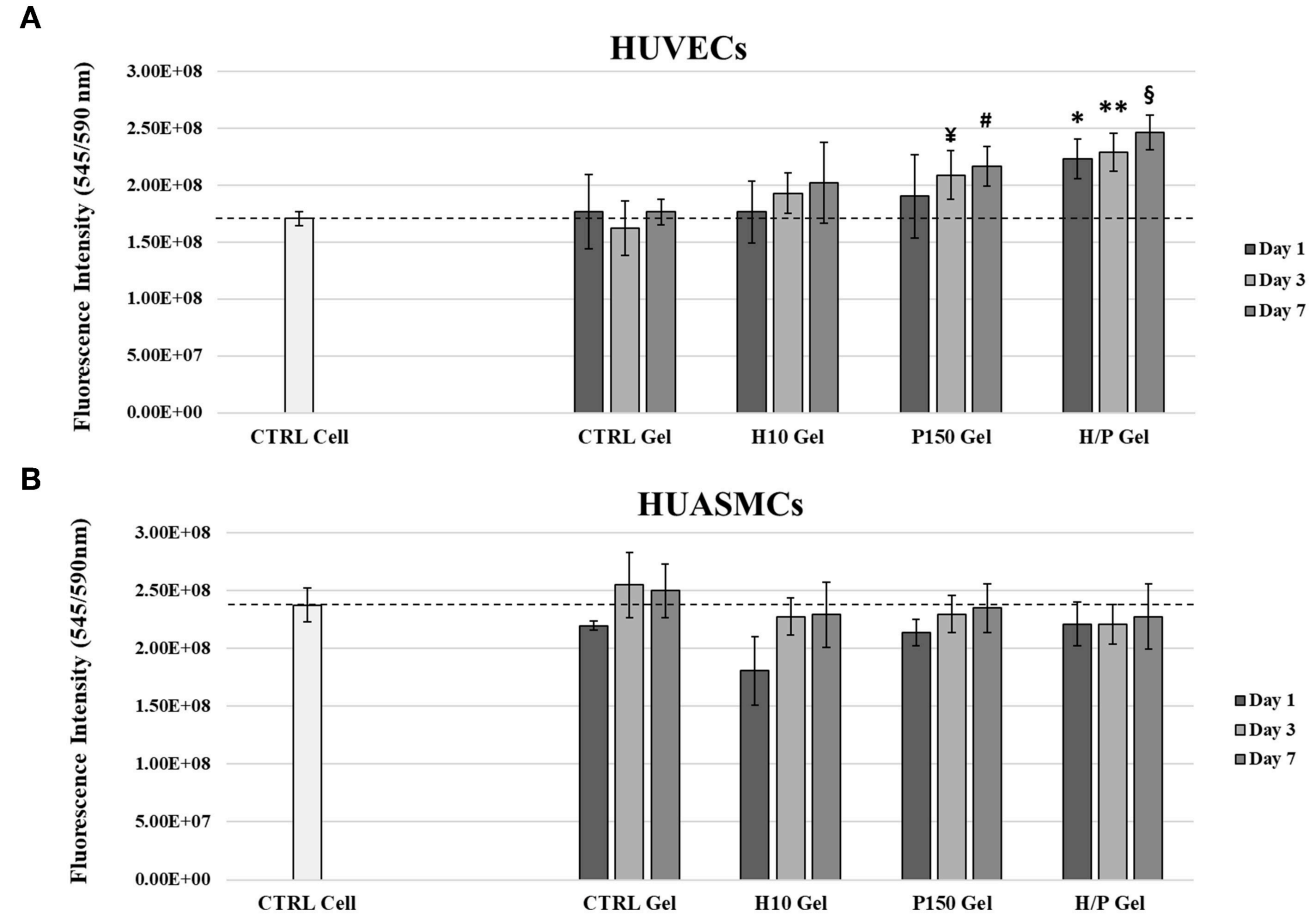
Indirect Viability Assay. HUVECs and HUASMCs were treated with conditioned medium collected after 1, 3, and 7 days of incubation with the following collagen gel conditions: control collagen gel (CTRL Gel); collagen gel with 10 μg/ml of heparin (H10 gel); collagen gel with 150 ng/ml of PTN (P150 gel); collagen gel containing 10 μg/ml of heparin and 150 ng/ml of PTN (H/P gel). Cell viability was measured after 24 h by means of a resazurin salt solution assay. **(A)** The graphic shows the relative viability ± SD recorded from HUVECs treated with the different experimental conditions. ^*^p < 0.01 vs. Day 1 CTRL Cell, CTRL Gel and H10 gel; ^**^p < 0.001 vs. Day 3 CTRL Cell and CTRL Gel; ^¥^*p* < 0.01 vs. Day 3 CTRL Gel; ^#^*p* < 0.05 vs. Day 7 CTRL Cell and CTRL Gel; ^§^*p* < 0.001 vs. Day 7 CTRL Cell and CTRL Gel. **(B)** The graphic shows the mean fluorescence ± SD recorded from HUASMCs treated with the different conditions.

Regarding the ECs (Figure 3A), viability test has shown that after 1 day of incubation, the conditioned medium collected after 1 day from the H/P gel condition (2.23 ± 0.17)E^8^ was able to significantly increase the viability compared to the CTRL Cell (1.71 ± 0.06)E^8^ (*p* < 0.01), the CTRL Gel (1.77 ± 0.33)E^8^ (*p* < 0.01), and the H10 gel (1.76 ± 0.30)E^8^ (*p* < 0.01) conditions. For the 3 days conditioned media, the P150 gel condition (2.09 ± 0.22)E^8^ was able to significantly increase the ECs viability against the CTRL Gel (1.62 ± 0.24)E^8^ (*p* < 0.01). Again, the H/P gel (2.29 ± 0.17)E^8^ was able to significantly increase the viability of the HUVECs compared to CTRL Cell (*p* < 0.001) and CTRL gel (*p* < 0.001). With the 7 days conditioned media, both P150 gel (2.17 ± 0.18)E^8^ and H/P gel (2.46 ± 0.15) conditions were able to significantly increase cells viability compared to the CTRL Cell (*p* < 0.05 vs. P150 gel, *p* < 0.001 vs. H/P gel) and CTRL Gel conditions (1.76 ± 0.11)E^8^ (*p* < 0.05 vs. P150 gel and *p* < 0.001 vs. H/P gel).

The indirect viability assay performed on SMCs has shown that, regardless of the time point at which the conditioned media were collected from the different collagen gel conditions, no significant change was observed in between the CTRL Cell and CTRL Gel conditions and the modified collagen gels (Figure 3B).

### Direct Viability Assay

**Figure 4:**
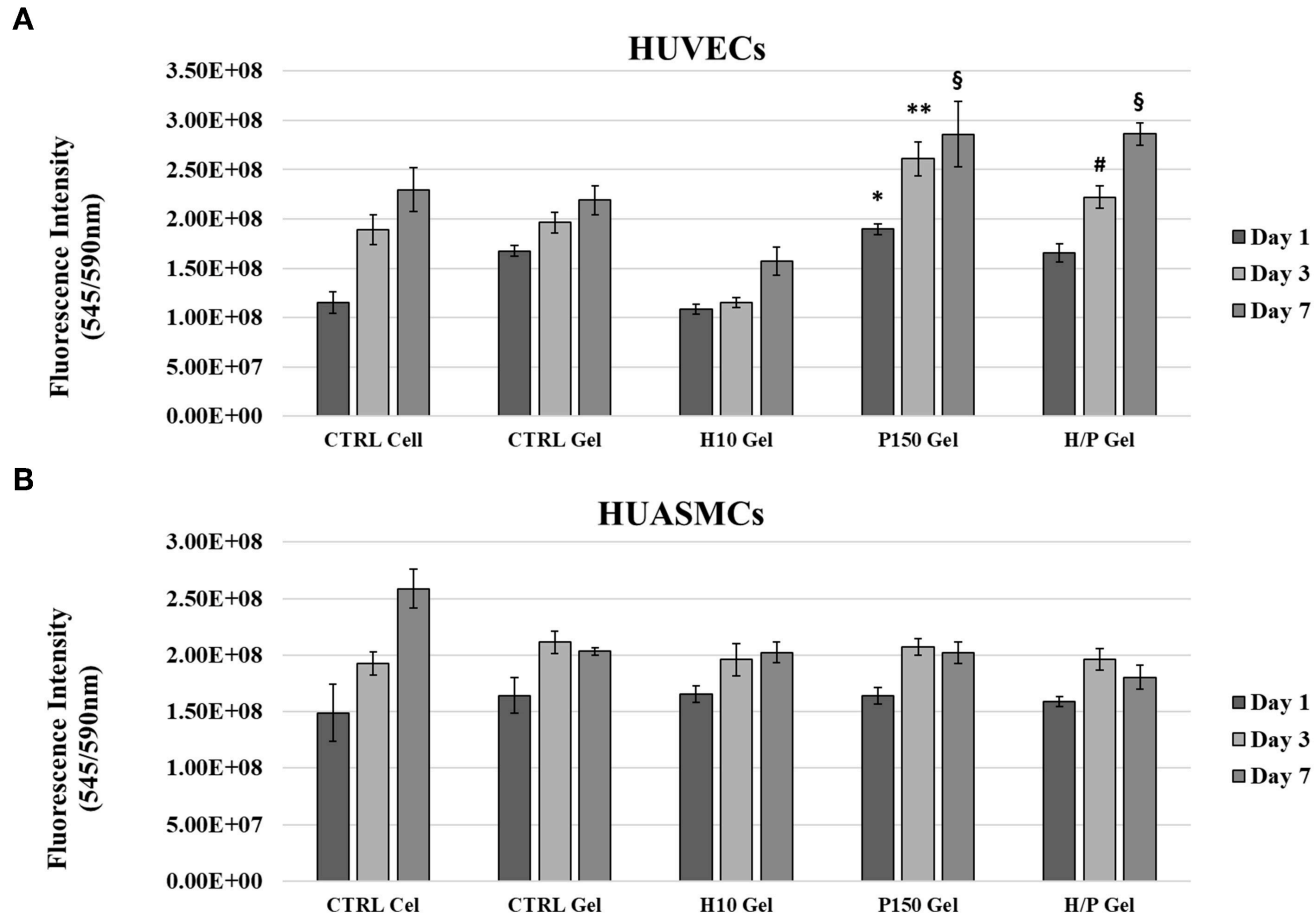
Direct Viability Assay. HUVECs and HUASMCs were directly seeded on the following collagen gel conditions: control collagen gel (CTRL Gel); collagen gel with 10 μg/ml of heparin (H10 gel); collagen gel with 150 ng/ml of PTN (P150 gel); collagen gel containing 10 μg/ml of heparin and 150 ng/ml of PTN (H/P gel). Cell viability was measured after 1, 2, and 7 days by means of a resazurin salt solution assay. **(A)** The graphic shows the mean fluorescence ± SD recorded from HUVECs treated with the different experimental conditions. ^*^p < 0.01 vs. Day 1 CTRL Cell and H10 gel; ^**^p < 0.001 vs. Day 3 CTRL Cell, CTRL Gel and H10 gel; ^#^*p* < 0.01 vs. Day 3 CTRL Cell, CTRL Gel and H10 gel; ^§^*p* < 0.01 vs. Day 7 CTRL Cell, CTRL Gel, and H10 gel. **(B)** The graphic shows the mean fluorescence ± SD recorded from HUASMCs treated with the different experimental conditions.

Direct viability tests have been performed on ECs and SMCs directly seeded on the different collagen gel conditions to evaluate the direct effects of the PTN present in the gels. After 1 day of incubation, HUVECs seeded on the P150 gels (1.90 ± 0.05)E^8^ showed a significant increased viability compared to the CTRL Cell (1.15 ± 0.11)E^8^ (*p* < 0.01) and to cells seeded on the H10 gels (1.08 ± 0.05)E^8^ (*p* < 0.01). After 3 days of incubation, both P150 gels (2.61 ± 0.17)E^8^ an H/P gels (2.22 ± 0.11)E^8^ were able to significantly increase the viability of the seeded HUVECs compared to the CTRL Cell (1.89 ± 0.20)E^8^ (*p* < 0.001 vs. P150 gel and *p* < 0.01 vs. H/P gel), CTRL gel (1.96 ± 0.11)E^8^ (*p* < 0.001 vs. P150 gel and *p* < 0.01 vs. H/P gel), and the H10 gel (1.15 ± 0.05)E^8^ (*p* < 0,001 vs. P150 gel and *p* < 0.01 vs. H/P gel). Finally, both P150 gels (2.86 ± 0.33)E^8^ an H/P gels (2.86 ± 0.11)E^8^ were able to significantly increase the viability of the seeded huvec compared to the CTRL Cell (2.30 ± 0.22)E^8^ (*p* < 0.001 vs. P150 gels and H/P gel), CTRL gels (2.19 ± 0.15)E^8^ (*p* < 0.001 vs. P150 gels and H/P gel), and H10 gels (1.57 ± 0.14)E^8^ (*p* < 0.001 vs. P150 gels and H/P gels) (Figure 4A).

Regarding the direct viability tests performed on the SMCs, as for the indirect tests, no significant difference was shown between the different experimental conditions (Figure 4B).

### Migration Assay

**Figure 5:**
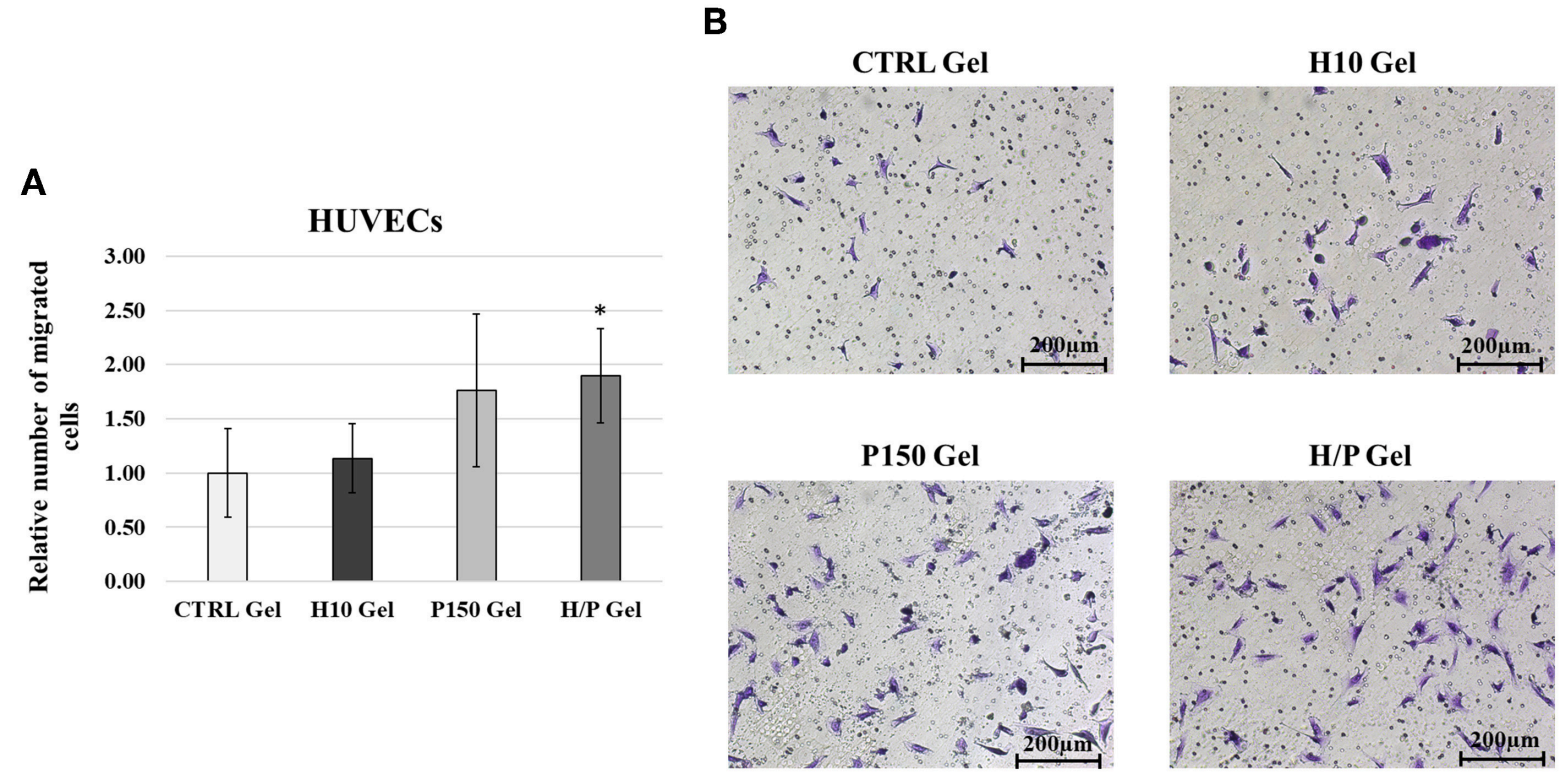
HUVECs Transwell Migration Assay. **(A)** Quantitative analysis of migrated HUVECs expressed as mean ± SD of number of migrated cells per field. Results have been normalized against the CTRL Gel condition. ^*^p < 0.01 vs. CTRL gel and H10 gel. **(B)** Representative images are showing the Crystal Violet staining of the migrated HUVECs for the four conditions tested: CTRL, H10, P150, and H/P gels. Images were taken at a 10X magnification.

EC migration was analyzed using the Transwell migration Assay. After 24 h of incubation, the H/P gel was able to induce a significant higher migration (1.90 ± 0.44) compared to the CTRL gel (1.00 ± 0.41; *p* < 0.001) and the H10 gel (1.14 ± 0.32; *p* < 0.001) (Figure 5).

**Figure 6:**
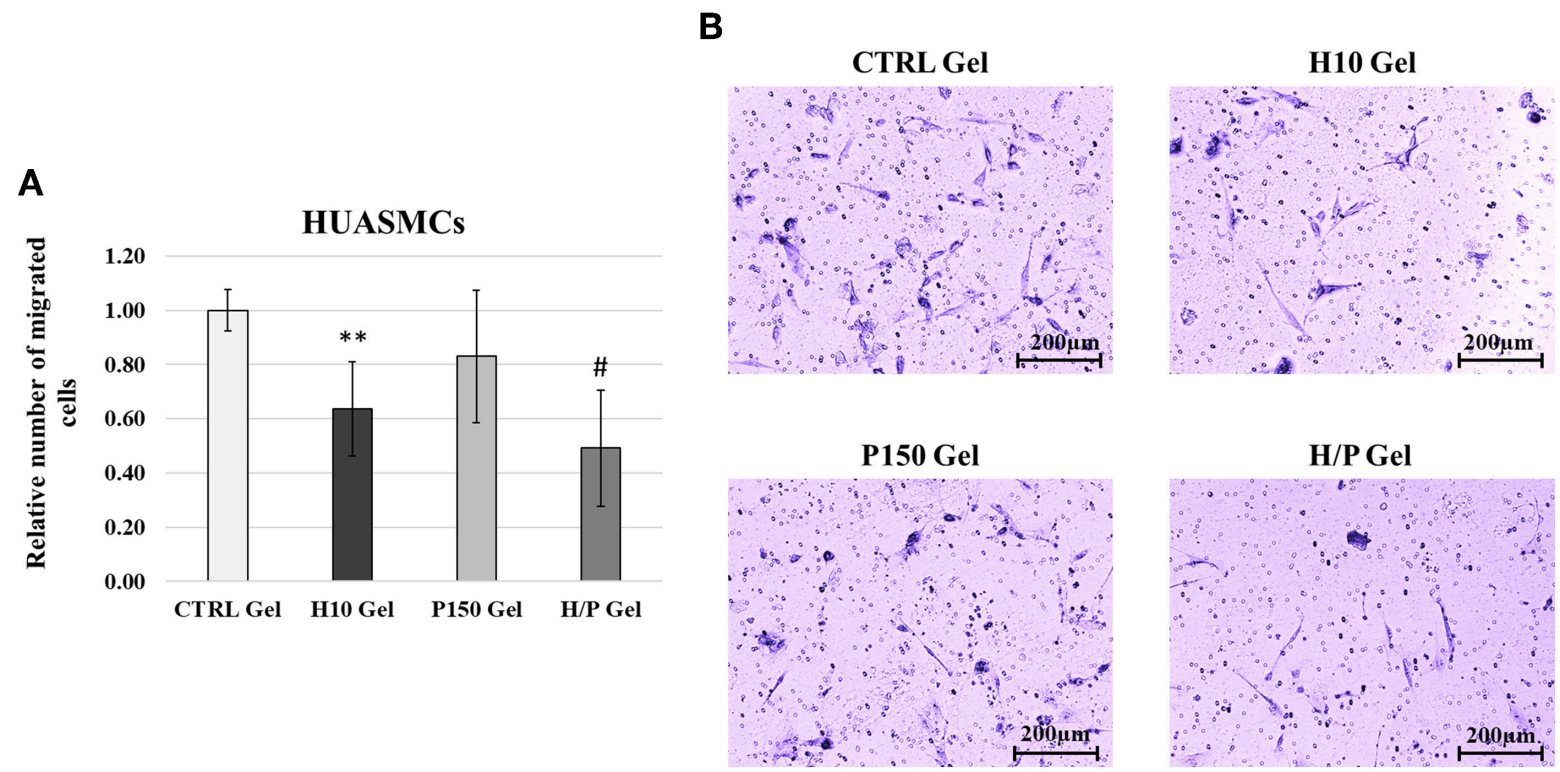
HUASMCs Transwell Migration Assay. **(A)** Quantitative analysis of migrated HUASMCs expressed as mean ± SD of number of migrated cells per field. Results have been normalized against the CTRL Gel condition. ^**^p < 0.01 vs. CTRL gel; ^#^*p* < 0.001 vs. CTRL gel and P150 gel. **(B)** Representative images are showing the Crystal Violet staining of the migrated HUASMCs for the four conditions tested: CTRL, H10, P150, and H/P gels. Images were taken at a 10X magnification.

Concerning the SMCs migration, the Transwell Assay (Figure 6) showed that after 24 h of incubation, the H10 gel were able to significantly inhibit the HUASMCs migration (0.64 ± 0.18) compared to the CTRL gel (*p* < 0.01). Moreover, in presence of the H/P gel the migration of the HUASMCs (0.49 ± 0.21) was also significantly lower if compared to the CTRL gel (*p* < 0.001) and the P150 gel (0.83 ± 0.24; *p* < 0.001).

### Hemocompatibility Assay

**Figure 7:**
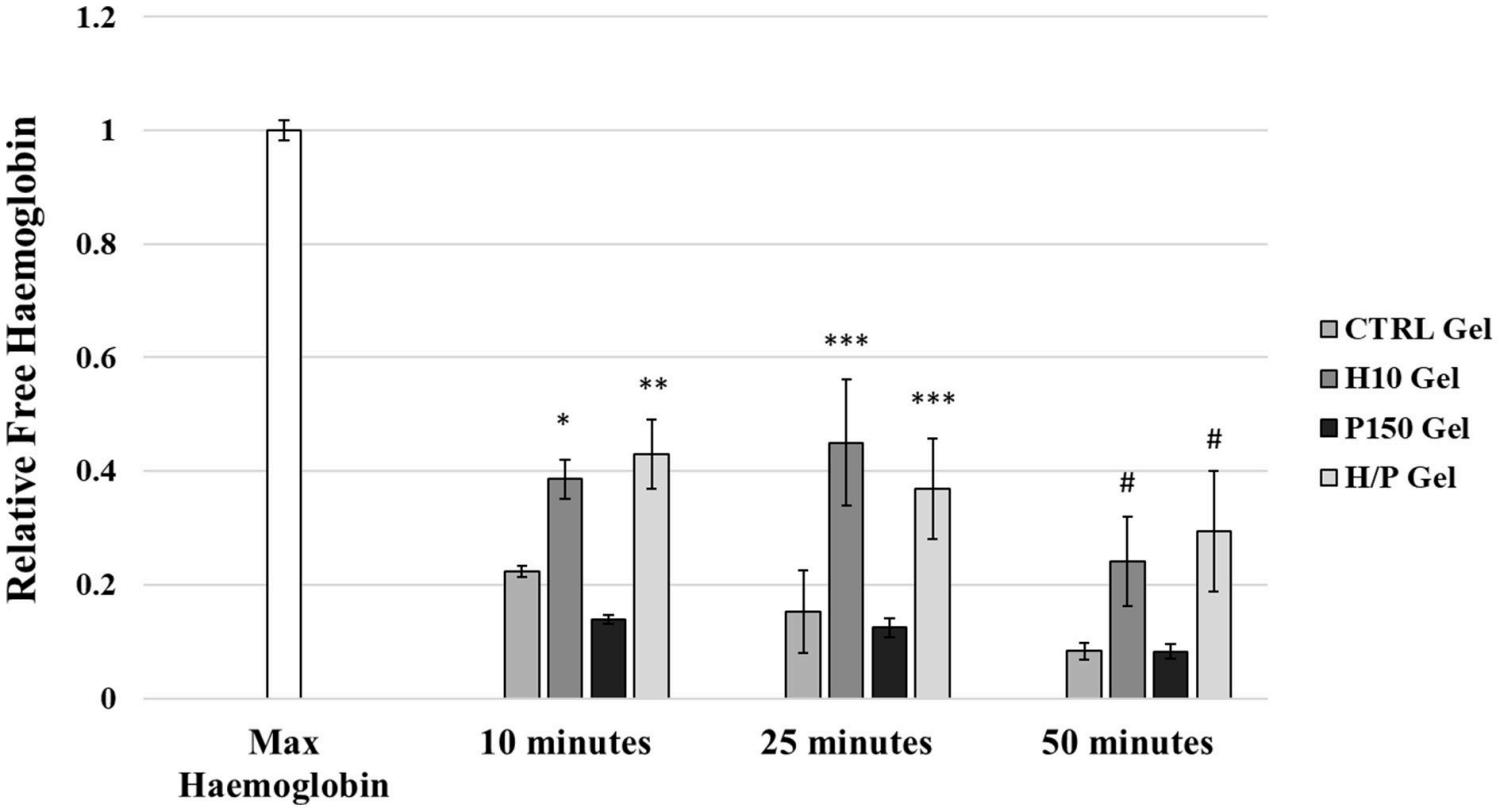
Hemocompatibility Test. Whole human blood was put in contact with the following collagen gel conditions: control collagen gel (CTRL Gel); collagen gel with 10 μg/ml of heparin (H10 gel); collagen gel with 150 ng/ml of PTN (P150 gel); collagen gel containing 10 μg/ml of heparin and 150 ng/ml of PTN (H/P gel). Blood was incubated with the gels for 10, 25, and 50 min. At each time point blood was solubilized and absorbance was recorded at 540 nm. The graphic shows the relative free hemoglobin ± SD. ^*^p < 0.001 vs. 10 min P150 gel; ^**^p < 0.05 vs. 10 min CTRL gel and *p* < 0.001 vs. 10 min P150 gel; ^***^p < 0.001 vs. 25 min CTRL gel and P150 gel; ^#^*p* < 0.001 vs. 50 min CTRL gel and P150 gel.

The hemocompatibility of the different collagen gel formulation has been tested using the hemoglobin free methodology. Absorbance at a wave length specific for hemoglobin (540 nm) was measured after the blood was incubated for 10, 25, and 50 min with the gels (Figure 7). After 10 min, both the gel formulation containing heparin, the H10 gel (0.39 ± 0.03) and the H/P gel (0.43 ± 0.06) where able to significantly increase the amount of free hemoglobin, hence the hemocompatibility, compared to both the CTRL gel (0.22 ± 0.01; *p* < 0.05 vs. H/P gel) and P150 gel (0.14 ± 0.01; *p* < 0.001 vs. H10 gel and H/P gel). The same behavior was observed after 25 min of incubation: both the H10 gel (0.45 ± 0.11) and the H/P gel (0.37 ± 0.09) were able to significantly increase the hemocompatibility compared to the CTRL gel (0.15 ± 0.07; *p* < 0.001 vs. H10 gel and H/P gel) and P150 gel (0.12 ± 0.02; *p* < 0.001 vs. H10 gel and H/P gel). Again, after 50 min of incubation, the H10 gel (0.24 ± 0.08) and the H/P gel (0.29 ± 0.11) significantly increase the measured free hemoglobin compared to the CTRL gel (0.08 ± 0.01; *p* < 0.001 vs. H10 gel and H/P gel) and the P150 gel (0.08 ± 0.01; *p* < 0.001 vs. H10 gel and H/P gel).

## Discussion

An effective and fast re-endothelialization is of crucial importance to guarantee the clinical patency of polymeric vascular graft (Bordenave et al., 2005). The formation of confluent endothelial coverage has the benefit to speed up and improve the integration of the implanted graft, effectively shortening the healing time (van der Zijpp et al., 2003). Moreover, graft endothelialization has the benefit of limiting the insurgence of adverse processes like in-graft thrombosis and neo-intima hyperplasia (Haruguchi and Teraoka, 2003). These two conditions, as of today, represent a major concern hampering the performances of synthetic vascular graft in the clinical practice. The modification of the luminal surface of synthetic graft with natural components of the vascular extracellular matrix (ECM), such as collagen, has been already used to provide receptor-ligand binding sites for ECs on the graft surface. Another important factor in promoting ECs adhesion and proliferation on the implanted graft is the use of biological signaling. This represents a crucial point in cell-driven tissue regeneration. However, the concentration of biological molecules must be fine-tuned in order to accomplish the desired effects, thus the need to release these molecules in a controlled way. Therefore, the development of collagen-based drug delivery systems, in which specific non-covalent interactions are used to stabilize small molecule and protein-based drugs, immobilize them within the collagen scaffold and to control their release for biomedical applications, have been widely developed (Wallace and Rosenblatt, 2003). These systems help in protecting the biological activity of the loaded molecules while slowing their diffusion from collagen scaffolds, providing optimal effects on the targeted vascular cells. The use of heparin to control the release of therapeutic agents from collagen scaffolds has been widely studied, due to its ability to sequester, stabilize, and protect growth factors and cytokines (Sakiyama-Elbert, 2014). In this study, a drug delivery system for PTN, a known pro-angiogenetic factor, is presented as a potential strategy to induce in-graft migration and proliferation of ECs. The release system is based on a Type 1 collagen hydrogel, chosen for his favorable biological properties. The hydrogel has been further modified with the addition of heparin to help controlling the release of the loaded PTN over time and to confer anticoagulant properties to the system. The mechanical properties of the modified gels have been tested, along with the biological performances. To do so, the different gel compositions have been tested for cell viability and migration on both ECs and SMCs, in order to evaluate their effects on cell behaviors.

The effect of the addition of heparin to collagen gels has been studied for long time (Obrink, 1973). Heparin was reported to alter the structure of the collagen fibers, potentially leading to changes in the mechanical and biological properties of the modified collagen gels (Guidry and Grinnell, 1987; Salchert et al., 2004). Mechanical tests leading to the equilibrium elastic modulus have shown how the addition of 10 μg/ml of heparin, H10 gels, does not alter in a significant way the mechanical properties of the CTRL gels (Figure 1A). Moreover, the immunofluorescence staining for the type 1 collagen fibers shows how the H10 gels presents an unaltered fibers structure, further demonstrating the absence of negative effects of heparin on the collagen gel structure (Figure 1B). The concentration chosen to modify the collagen gel, besides showing the best results in terms of structure preservation compared to other concentration tested (data not shown), is in accordance with pre-existing data present in literature (Johnson et al., 2010).

The release of growth factors and cytokine from heparinized collagen gels is dependent on diffusion from the collagen matrix and the binding affinity to heparin, whereas for simple collagen gels the release rely mainly on the diffusion from the gel (Lee et al., 2004). The analysis of the release of PTN from the unmodified (P150 gels) and heparin-modified gels (H/P gels) shed light on how the addition of heparin was able to induce a more controlled, sustained release of PTN over the time period studied. The amount of PTN released during the first week by the H/P gels is significantly lower compared to the P150 gels. Moreover, the amount of PTN released by the H/P gels increase until the fourteenth day, whereas a significant drop in release is notable with the P150 gels. The results here obtained are in accordance with previous observation made with the use of heparin to modulate the release of several growth factor (Lee et al., 2004; Yang et al., 2012; Hao et al., 2018), implying a direct effect of heparin in controlling the release of PTN from the collagen gels. Moreover, as shown in Figure 2B, the addition of heparin to collagen gels resulted effective in decreasing the initial burst of PTN released, as with previously described results where heparin successfully controlled the release of growth factors from heparinized scaffolds (Lee et al., 2007; Liu et al., 2016).

PTN is known for is beneficial effects exerted on the cardiovascular system, and especially on ECs. In fact, it has been shown to be a neovasculogenesis inducer (Christman et al., 2005), a potent pro-angiogenetic factor (Besse et al., 2013) and to be able to effectively differentiate mononuclear cells into functional ECs (Palmieri et al., 2015). Moreover, as described in our previous work (Copes et al., 2019), PTN can induce significant effects on viability, migration and repair ability of ECs. The results hereby presented show how the incorporation of PTN in the Heparin-Collagen gel delivery system does not altered the beneficial effects toward the ECs associated to PTN. Regarding the HUVECs directly seeded onto the different collagen gel formulations, the presence of PTN was able to induce a significant increase in cell viability compared to the conditions without it. Moreover, while in presence of the sole heparin (H10 gel condition) a decrease in ECs viability is appreciable, in the H/P gels no negative effects are detectable, showing a behavior similar to the P150 gels (Figure 4A), displaying the ability of the added PTN to “mask” the effect of heparin. Those results are in accordance with our previous findings about PTN mitogenic effects on ECs. Regarding the indirect viability tests (Figure 3A), again PTN was able to significantly increase the treated HUVECs viability compared to the control conditions. Of interest, the released PTN from the H/P gels seems to exert better effects compared to the one obtained from the P150 gels. This difference in efficacity could be explained by the presence of heparin in the H/P gels: heparin has been demonstrated to be able to stabilize and preserve the structure, thus the function of several growth factors and cytokine (Nissen et al., 1999; Sakiyama-Elbert, 2014). The binding of heparin to growth factor has been also demonstrated to be able to increase the efficacy of the bounded factors (Ornitz et al., 1992). Considering the high binding affinity between PTN and heparin, a protective action of the latter on the released PTN could be at the base of the H/P gel released PTN effects toward the treated ECs. The results obtained from the migration assay show a response from the ECs similar to the one obtained for the viability assay. As shown in Figure 5, the H/P gels were able to induce a significant increase in the migration of the treated HUVECs compare to control conditions, confirming the beneficial effects of PTN on ECs migration (Lampropoulou et al., 2018; Copes et al., 2019). Interesting, contrary to the H/P gels, the effect exerted by the P150 gels resulted being not significantly higher compared to the control conditions, further validating the hypothesis of a protective/enhancing effect of heparin on PTN activity toward ECs.

Regarding the effects of PTN on the viability and migration of SMCs, the results obtained gave a different picture. In fact, both the indirect and direct cell viability test (Figures 3B, 4B) show how the presence of PTN does not induce any significant effect on the viability of treated SMCs, contrary to the results presented by Brewster et al. (2004), where the treatments with a chimeric PTN fusion protein induced an increase in the viability of SMCs, thus suggesting a non-specificity of PTN for SMCs. The results of the migration assay performed on the HUASMCs (Figure 6), show how with the H10 and H/P gels, meaning in presence of heparin, the migration ability of the SMCs was significantly inhibited compared to the CTRL and P150 gels. The inhibitory effects of heparin on SMCs viability, proliferation, and migration ability (Castellot et al., 1985; Stewart et al., 2012) along with their mechanism (Young, 2008; Gilotti et al., 2014) are well known. This, along with the results hereby presented, suggests a role for the heparin in the inhibitory effects observed on the treated HUASMCs. Moreover, since heparin added to the gels is not immobilized in the collagen matrix, this may account for a portion of the added heparin to be released along with PTN, thus explaining the inhibitory effects observed on SMCs migration and the aforementioned protective/enhancing effect on PTN toward ECs. Altogether, these findings demonstrate how are system could be useful for an application in vascular graft functionalization, showing pro-endothelialization properties and inhibitory effects on SMCs, two of the most sought-after effects for vascular biomaterials.

The effects of heparin as an anticoagulant factor are well known and characterized (Gray et al., 2012), as is its use in tissue engineering to confer anticoagulation properties (Liang and Kiick, 2014). In accordance to the data present in literature, the hemocompatibility of the gels containing heparin, in particular in the H/P gels, was significantly increase compared to the CTRL and P150 gels, suggesting a potential in limiting the formation of thrombosis in an *in vivo* application (Figure 7). These results fall in accordance with the existing literature, where the use of heparin to modify collagen scaffolds to enhance their hemocompatibility properties has already been investigated with promising results (Keuren et al., 2004). Moreover, the previously described effects of the addition of heparin on the release of PTN and the preservation of its beneficial effects, further support the possible application in vascular medicine of this drug delivery system.

## Conclusions

The obtained results show how the addition of heparin to a type I collagen gel can control over time the release of PTN, without altering the gel properties while limiting the thrombogenicity of the modified gels. The added PTN, moreover, is able to exert beneficial ECs-specific effects on cell viability and migration while not affecting SMCs behavior. In conclusion, the PTN-heparin-modified collagen gels here proposed can represent an added value for their use in vascular medicine, being able to improve the biological performance and integration of vascular grafts.

## Author Contributions

FC, FB, and DM conceived the presented idea. FC, PC, DM, and FB conceived and planned the experiments. FC carried out the experiments with the help of CL and DP. FC, PC, CL, and DP contributed to the interpretation of the results. FC wrote the manuscript with substantial help from PC and CL and in consultation with FB and DM. FB and DM supervised the project.

### Conflict of Interest Statement

The authors declare that the research was conducted in the absence of any commercial or financial relationships that could be construed as a potential conflict of interest.

## Footnotes

^1^*WHO Top 10 Causes of Death* [Online]. Available online at: http://www.who.int/gho/mortality_burden_disease/causes_death/top_10/en/ World Health Organization (accessed January 28, 2019).

^2^Association, A. H. *Atherosclerosis* [Online]. Available online at: http://www.heart.org/HEARTORG/Conditions/Cholesterol/WhyCholesterolMatters/Atherosclerosis_UCM_305564_Article.jsp#.WPorq4jhDIU.heart.org/HEARTORG/Conditions/Cholesterol/WhyCholesterolMatters/Atherosclerosis_UCM_305564_Article.jsp#.WPorq4jhDIU (accessed May 8, 2017).

## References

[B1] Adipurnama I. Yang M. C. Ciach T. Butruk-Raszeja B. (2016). Surface modification and endothelialization of polyurethane for vascular tissue engineering applications: a review. Biomater. Sci. 5, 22–37. 10.1039/C6BM00618C 27942617

[B2] Avci-Adali M. Perle N. Ziemer G. Wendel H. P. (2011). Current concepts and new developments for autologous *in vivo* endothelialisation of biomaterials for intravascular applications. Eur. Cell Mater. 21, 157–176. 10.22203/eCM.v021a13 21312162

[B3] Besse S. Comte R. Fréchault S. Courty J. Joël de L. Delbé J. (2013). Pleiotrophin promotes capillary-like sprouting from senescent aortic rings. Cytokine 62, 44–47. 10.1016/j.cyto.2013.02.002 23481101

[B4] Boccafoschi F. Rajan N. Habermehl J. Mantovani D. (2007). Preparation and characterization of a scaffold for vascular tissue engineering by direct-assembling of collagen and cells in a cylindrical geometry. Macromol. Biosci. 7, 719–726. 10.1002/mabi.200600242 17457943

[B5] Bordenave L. Fernandez P. Rémy-Zolghadri M. Villars S. Daculsi R. Midy D. (2005). *In vitro* endothelialized ePTFE prostheses: clinical update 20 years after the first realization. Clin. Hemorheol. Microcirc. 33, 227–234. 16215288

[B6] Bradley W. G. Wilkes G. L. (1977). Some mechanical property considerations of reconstituted collagen for drug release supports. Biomater. Med. Devices Artif. Organs. 5, 159–175. 10.3109/10731197709118671 560220

[B7] Brewster L. P. Brey E. M. Tassiopoulos A. K. Xue L. Maddox E. Armistead D.. (2004). Heparin-independent mitogenicity in an endothelial and smooth muscle cell chimeric growth factor (S130K-HBGAM). Am. J. Surg. 188, 575–579. 10.1016/j.amjsurg.2004.07.012 15546573

[B8] Bush H. L. J. (1989). Mechanism of Graft Failure. J. Vasc. Surg. 9, 392–394. 10.1016/0741-5214(89)90073-6

[B9] Castellot J. J. Cochran D. L. Karnovsky M. J. (1985). Effect of heparin on vascular smooth muscle cells. I. Cell metabolism. J. Cell Physiol. 124, 21–28. 10.1002/jcp.1041240105 4044651

[B10] Chester J. F. (2002). The causes of synthetic vascular graft failure. Ann. Coll. Surg. HK 6, 97–101. 10.1046/j.1442-2034.2002.00149.x

[B11] Chiu L. L. Radisic M. Vunjak-Novakovic G. (2010). Bioactive scaffolds for engineering vascularized cardiac tissues. Macromol. Biosci. 10, 1286–1301. 10.1002/mabi.201000202 20857391 PMC3627738

[B12] Chlupác J. Filová E. Bacáková L. (2009). Blood vessel replacement: 50 years of development and tissue engineering paradigms in vascular surgery. Physiol. Res. 58(Suppl. 2), S119–139. 20131930 10.33549/physiolres.931918

[B13] Christman K. L. Fang Q. Kim A. J. Sievers R. E. Fok H. H. Candia A. F.. (2005). Pleiotrophin induces formation of functional neovasculature *in vivo*. Biochem. Biophys. Res. Commun. 332, 1146–1152. 10.1016/j.bbrc.2005.04.174 15949466

[B14] Copes F. Ramella M. Fusaro L. Mantovani D. Cannas M. Boccafoschi F. (2019). Pleiotrophin: analysis of the endothelialisation potential. Adv. Med. Sci. 64, 144–151. 10.1016/j.advms.2018.08.007 30660899

[B15] De Visscher G. Mesure L. Meuris B. Ivanova A. Flameng W. (2012). Improved endothelialization and reduced thrombosis by coating a synthetic vascular graft with fibronectin and stem cell homing factor SDF-1alpha. Acta Biomater. 8, 1330–1338. 10.1016/j.actbio.2011.09.016 21964214

[B16] Deuel T. F. Zhang N. Yeh H. J. Silos-Santiago I. Wang Z. Y. (2002). Pleiotrophin: a cytokine with diverse functions and a novel signaling pathway. Arch. Biochem. Biophys. 397, 162–171. 10.1006/abbi.2001.2705 11795867

[B17] Gelse K. Pöschl E. Aigner T. (2003). Collagens–structure, function, and biosynthesis. Adv. Drug Deliv. Rev. 55, 1531–1546. 10.1016/j.addr.2003.08.002 14623400

[B18] Gilotti A. C. Nimlamool W. Pugh R. Slee J. B. Barthol T. C. Miller E. A.. (2014). Heparin responses in vascular smooth muscle cells involve cGMP-dependent protein kinase (PKG). J. Cell Physiol. 229, 2142–2152. 10.1002/jcp.24677 24911927 PMC4149598

[B19] Gray E. Hogwood J. Mulloy B. (2012). The anticoagulant and antithrombotic mechanisms of heparin. Handb. Exp. Pharmacol. 207, 43–61. 10.1007/978-3-642-23056-1_3 22566220

[B20] Greisler H. P. (1996). Growth factor release from vascular grafts. J. Control Release 39, 267–280. 10.1016/0168-3659(95)00159-X

[B21] Guidry C. Grinnell F. (1987). Heparin modulates the organization of hydrated collagen gels and inhibits gel contraction by fibroblasts. J. Cell Biol. 104, 1097–1103. 10.1083/jcb.104.4.1097 3558481 PMC2114446

[B22] Habermehl J. Skopinska J. Boccafoschi F. Sionkowska A. Kaczmarek H. Laroche G.. (2005). Preparation of ready-to-use, stockable and reconstituted collagen. Macromol. Biosci. 5, 821–828. 10.1002/mabi.200500102 16121339

[B23] Hao W. Han J. Chu Y. Huang L. Zhuang Y. Sun J.. (2018). Collagen/heparin bi-affinity multilayer modified collagen scaffolds for controlled bFGF release to improve angiogenesis *in vivo*. Macromol. Biosci. 18:e1800086. 10.1002/mabi.201800086 30160040

[B24] Haruguchi H. Teraoka S. (2003). Intimal hyperplasia and hemodynamic factors in arterial bypass and arteriovenous grafts: a review. J. Artif. Organs. 6, 227–235. 10.1007/s10047-003-0232-x 14691664

[B25] Johnson M. R. Boerckel J. D. Dupont K. M. Cool S. M. Guldberg R. E. (2010). Integration of heparin in collagen delivery system for BMP-2 induced bone repair, in 56th Annual Meeting of the Orthopedic Research Society (New Orleans, LA: Orthopaedic Research Society).

[B26] Keuren J. F. Wielders S. J. Driessen A. Verhoeven M. Hendriks M. Lindhout T. (2004). Covalently-bound heparin makes collagen thromboresistant. Arterioscler. Thromb. Vasc. Biol. 24, 613–617. 10.1161/01.ATV.0000116026.18945.66 14707039

[B27] Koch S. Yao C. Grieb G. Prével P. Noah E. M. Steffens G. C. (2006). Enhancing angiogenesis in collagen matrices by covalent incorporation of VEGF. J. Mater. Sci. Mater. Med. 17, 735–741. 10.1007/s10856-006-9684-x 16897166

[B28] Lampropoulou E. Logoviti I. Koutsioumpa M. Hatziapostolou M. Polytarchou C. Skandalis S. S.. (2018). Cyclin-dependent kinase 5 mediates pleiotrophin-induced endothelial cell migration. Sci. Rep. 8:5893. 10.1038/s41598-018-24326-x 29651006 PMC5897396

[B29] Lee C. H. Singla A. Lee Y. (2001). Biomedical applications of collagen. Int. J. Pharm. 221, 1–22. 10.1016/S0378-5173(01)00691-3 11397563

[B30] Lee H. Chung H. J. Park T. G. (2007). Perspectives on: local and sustained delivery of angiogenic growth factors. J. Bioact. Compat. Polym. 22, 89–114. 10.1177/0883911506073363

[B31] Lee K. W. Yoon J. J. Lee J. H. Kim S. Y. Jung H. J. Kim S. J.. (2004). Sustained release of vascular endothelial growth factor from calcium-induced alginate hydrogels reinforced by heparin and chitosan. Transplant. Proc. 36, 2464–2465. 10.1016/j.transproceed.2004.08.078 15561282

[B32] Liang Y. Kiick K. L. (2014). Heparin-functionalized polymeric biomaterials in tissue engineering and drug delivery applications. Acta Biomater. 10, 1588–1600. 10.1016/j.actbio.2013.07.031 23911941 PMC3937301

[B33] Liu Y. Deng L. Z. Sun H. P. Xu J. Y. Li Y. M. Xie X.. (2016). Sustained dual release of placental growth factor-2 and bone morphogenic protein-2 from heparin-based nanocomplexes for direct osteogenesis. Int. J. Nanomed. 11, 1147–1158. 10.2147/IJN.S100156 27042064 PMC4809329

[B34] Loy C. Meghezi S. Lévesque L. Pezzoli D. Kumra H. Reinhardt D.. (2016). A planar model of the vessel wall from cellularized-collagen scaffolds: focus on cell-matrix interactions in mono-, bi- and tri-culture models. Biomater. Sci. 5, 153–162. 10.1039/C6BM00643D 27918018

[B35] Lynn A. K. Yannas I. V. Bonfield W. (2004). Antigenicity and immunogenicity of collagen. J. Biomed. Mater. Res. B Appl. Biomater. 71, 343–354. 10.1002/jbm.b.30096 15386396

[B36] Martínez-González B. Reyes-Hernández C. G. Quiroga-Garza A. Rodríguez-Rodríguez V. E. Esparza-Hernández C. N. Elizondo-Omaña R. E.. (2017). Conduits used in coronary artery bypass grafting: a review of morphological studies. Ann. Thorac. Cardiovasc. Surg. 23, 55–65. 10.5761/atcs.ra.16-00178 28202895 PMC5422630

[B37] Montaño-Machado V. Chevallier P. Mantovani D. Pauthe E. (2015). On the potential for fibronectin/phosphorylcholine coatings on PTFE substrates to jointly modulate endothelial cell adhesion and hemocompatibility properties. Biomatter 5:e979679. 10.4161/21592535.2014.979679 25785369 PMC4581125

[B38] Nissen N. N. Shankar R. Gamelli R. L. Singh A. DiPietro L. A. (1999). Heparin and heparan sulphate protect basic fibroblast growth factor from non-enzymic glycosylation. Biochem. J. 338(Pt 3), 637–642. 10051433 PMC1220097

[B39] Obrink B. (1973). The influence of glycosaminoglycans on the formation of fibers from monomeric tropocollagen *in vitro*. Eur. J. Biochem. 34, 129–137. 10.1111/j.1432-1033.1973.tb02739.x 4267112

[B40] Ornitz D. M. Yayon A. Flanagan J. G. Svahn C. M. Levi E. Leder P. (1992). Heparin is required for cell-free binding of basic fibroblast growth factor to a soluble receptor and for mitogenesis in whole cells. Mol. Cell Biol. 12, 240–247. 10.1128/MCB.12.1.240 1309590 PMC364088

[B41] Palmieri D. Mura M. Mambrini S. Palombo D. (2015). Effects of Pleiotrophin on endothelial and inflammatory cells: pro-angiogenic and anti-inflammatory properties and potential role for vascular bio-prosthesis endothelialization. Adv. Med. Sci. 60, 287–293. 10.1016/j.advms.2015.05.003 26114799

[B42] Rabenstein D. L. (2002). Heparin and heparan sulfate: structure and function. Nat. Prod. Rep. 19, 312–331. 10.1039/b100916h 12137280

[B43] Rauvala H. (1989). An 18-kd heparin-binding protein of developing brain that is distinct from fibroblast growth factors. EMBO J. 8, 2933–2941. 10.1002/j.1460-2075.1989.tb08443.x 2583087 PMC401361

[B44] Ravi S. Chaikof E. L. (2010). Biomaterials for vascular tissue engineering. Regen. Med. 5, 107–120. 10.2217/rme.09.77 20017698 PMC2822541

[B45] Rojas-Mayorquín A. E. Ortuño-Sahagún D. (2017). Pleiotrophin, in Encyclopedia of Signaling Molecules, 2nd Edn. ed Choi S. (Cham: Springer).

[B46] Sakiyama-Elbert S. E. (2011). Drug Delivery via Heparin Conjugates, in Comprehensive Biomaterials. eds. Grainger D. W. Kirkpatrick C. J. (Elsevier Inc), 333–338.

[B47] Sakiyama-Elbert S. E. (2014). Incorporation of heparin into biomaterials. Acta Biomater. 10, 1581–1587. 10.1016/j.actbio.2013.08.045 24021232 PMC3949739

[B48] Salchert K. Streller U. Pompe T. Herold N. Grimmer M. Werner C. (2004). *In vitro* reconstitution of fibrillar collagen type I assemblies at reactive polymer surfaces. Biomacromolecules 5, 1340–1350. 10.1021/bm0499031 15244449

[B49] Springer M. L. (2006). A balancing act: therapeutic approaches for the modulation of angiogenesis. Curr. Opin. Investig. Drugs 7, 243–250. 16555684

[B50] Steffens G. C. Yao C. Prével P. Markowicz M. Schenck P. Noah E. M.. (2004). Modulation of angiogenic potential of collagen matrices by covalent incorporation of heparin and loading with vascular endothelial growth factor. Tissue Eng. 10, 1502–1509. 10.1089/ten.2004.10.1502 15588409

[B51] Stewart E. M. Liu X. Clark G. M. Kapsa R. M. Wallace G. G. (2012). Inhibition of smooth muscle cell adhesion and proliferation on heparin-doped polypyrrole. Acta Biomater. 8, 194–200. 10.1016/j.actbio.2011.07.029 21843664

[B52] van der Zijpp Y. J. Poot A. A. Feijen J. (2003). Endothelialization of small-diameter vascular prostheses. Arch. Physiol. Biochem. 111, 415–427. 10.3109/13813450312331342274 16026029

[B53] Wallace D. G. Rosenblatt J. (2003). Collagen gel systems for sustained delivery and tissue engineering. Adv. Drug Deliv. Rev. 55, 1631–1649. 10.1016/j.addr.2003.08.004 14623405

[B54] Yang H. S. La W. G. Cho Y. M. Shin W. Yeo G. D. Kim B. S. (2012). Comparison between heparin-conjugated fibrin and collagen sponge as bone morphogenetic protein-2 carriers for bone regeneration. Exp. Mol. Med. 44, 350–355. 10.3858/emm.2012.44.5.039 22322342 PMC3366328

[B55] Young E. (2008). The anti-inflammatory effects of heparin and related compounds. Thromb. Res. 122, 743–752. 10.1016/j.thromres.2006.10.026 17727922

